# Organic chemistry on surfaces: Direct cyclopropanation by dihalocarbene addition to vinyl terminated self-assembled monolayers (SAMs)

**DOI:** 10.3762/bjoc.10.307

**Published:** 2014-12-05

**Authors:** Malgorzata Adamkiewicz, David O’Hagan, Georg Hähner

**Affiliations:** 1 EaStCHEM School of Chemistry, University of St. Andrews, St. Andrews, Fife KY16 9ST, UK

**Keywords:** difluoro-, dichloro-, dibromomethylenecyclopropanes, dihalocarbenes, self-assembled monolayers, surface coating

## Abstract

C11-Vinyl-terminated self-assembled monolayers (SAMs) on silica surfaces are successfully modified in C–C bond forming reactions with dihalocarbenes to generate SAMs, terminated with dihalo- (fluoro, chloro, bromo) cyclopropane motifs with about 30% surface coverage.

## Introduction

**Figure 1:**
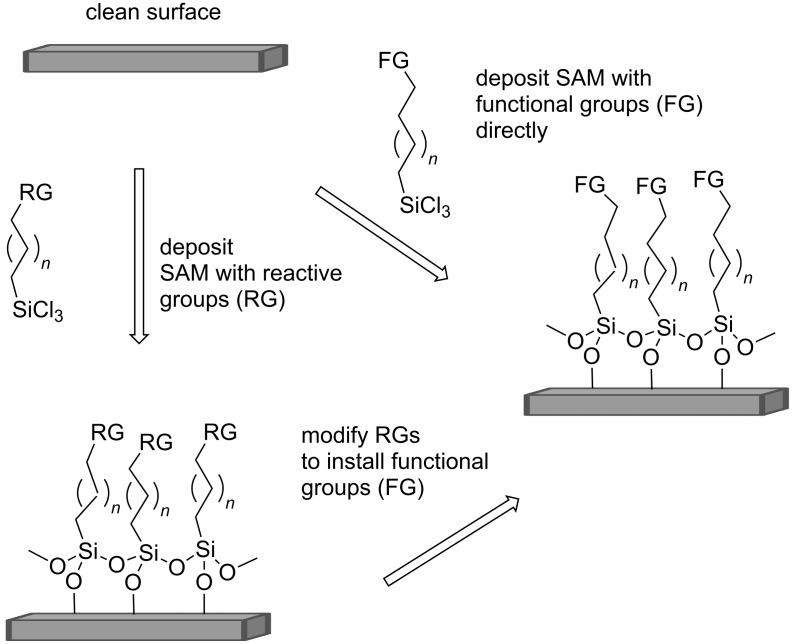
General strategies for incorporating functional groups (FGs) on the surface of self-assembled monolayers (SAMs), illustrated for alkylsilanes onto silica.

Self-assembled monolayers (SAMs) are increasingly being used as a means of surface modification to alter properties in a tuneable manner [1–3]. The major classes of SAMs are those with adsorbed long chain alkyl thiols on gold surfaces/nanoparticles [4–5], or long chain alkylsilanes on silica surfaces [6–7]. Two general approaches are taken to achieve surface modification as illustrated in Figure 1. The first involves incorporating pre-functionalised alkylsilanes/alkylthiols carrying functional groups (FG) to generate the SAM directly, whereas the second approach involves chemical modification of a pre-assembled monolayer carrying reactive groups (RG), as a means to introduce the SAM carrying the FGs [8]. Both approaches present challenges. In the former the desired functionality (FG) requires to be robust and orthogonal in reactivity to the chemistry involved in securing the substrate to the organic film (e.g., FG-Alkyl-SiCl_3_ and silicon substrate). In the latter chemical modification of the reactive groups of the pre-coated SAM has to be efficient enough such that a reasonable conversion can be obtained, with chemical specificity and lack of surface degradation. In this respect ‘click’ reactions have become attractive including azide–alkyne cycloadditions [9–10], Diels–Alder reactions [11–12], maleimide–thiol reactions [13], thiol–ene additions [14], and imine/oxime conjugations [15]. In this article we demonstrate that dihalocarbenes can be used to generate dihalocyclopropanes on olefin terminated SAMs.

We recently reported the formation of high quality vinyl-terminated SAMs generated from the vapour phase by adsorption of octadecyltrichlorosilanes onto silicon wafers [16]. With access to these SAMs it became an objective to explore functional group modification of the vinyl double bond. Carbon–carbon bond formation of vinyl-terminated SAMs has been demonstrated, e.g., through surface modification of radicals generated by C–O bond thermolysis [17] and in a more controlled sense via olefin cross metathesis/enyne metathesis [18] of mixed vinyl and acetylenyl-terminated SAMs followed by Diels–Alder modifications of the resultant dienes [19]. We are not aware however of straightforward carbene additions having been explored with olefin-terminated SAMs. The potential for product cyclopropanes offered a modification of limited steric impact, but if suitably substituted may be used to tune surface properties. Therefore dihalocyclopropanes emerged as an attractive controlled modification particularly as the precursor dihalocarbenes are relatively easily generated [20]. In this context we report dihalocyclopropanation of pre-assembled vinyl-terminated SAMs. Three dihalocarbene modifications were explored involving dibromo- (:CBr_2_), dichloro- (:CCl_2_) and difluoro- (:CF_2_) carbenes [21–23]. The resultant SAMs were analysed by X-ray photoelectron spectroscopy (XPS), contact angle goniometry, ellipsometry, and atomic force microscopy.

## Results and Discussion

**Figure 2:**
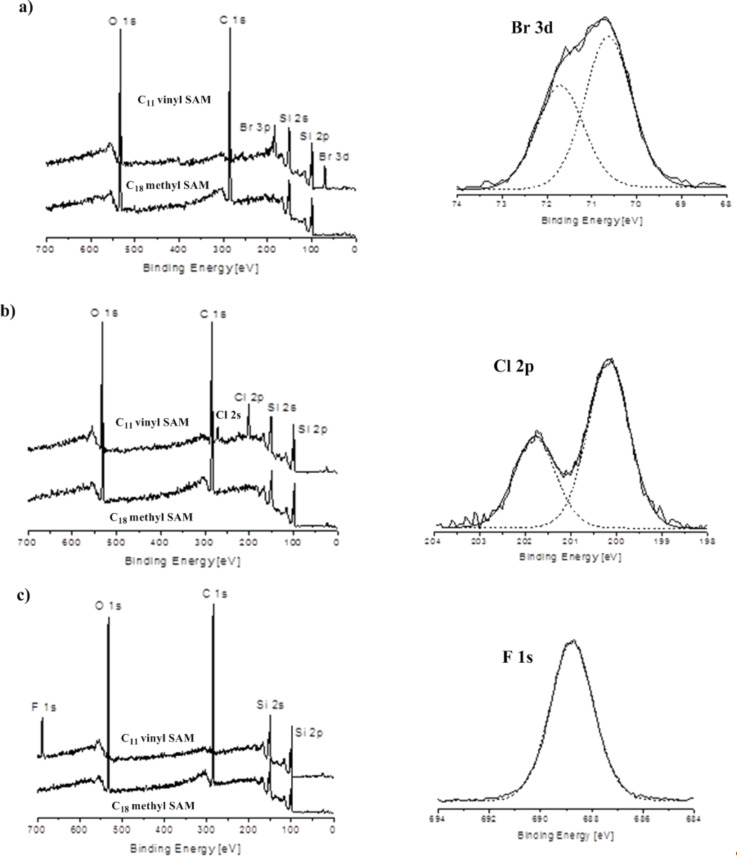
XPS scans after reactions with a) :CBr_2_; b) :CCl_2_ and c) :CF_2_. In each case the upper traces are scans of C_11_-vinyl SAMs, and the lower traces are C_18_-Me-terminated SAMs each treated with the relevant carbene reaction solution. The expanded regions on the right hand side, associated with each C_11_-vinyl SAM spectrum, show the key halide specific XPS signals.

After exposure to carbenes the vinyl-terminated SAMs were characterised by XPS, contact angle measurements and ellipsometry (see Supporting Information File 1). With XPS elements such as silicon, carbon and oxygen were expected in all cases [16]. In each case control reactions were also carried out on the C_18_-methyl (Me)-terminated SAMs, to ensure that only the vinyl group was responsible for surface reactivity. The resultant XPS analyses are shown for the vinyl-terminated SAMs for each carbene in Figure 2, and directly underneath, the lower traces illustrate the corresponding analyses after exposure of the carbene solutions to the C_18_-Me-terminated SAMs.

Figure 2a represents a surface after chemical modification with :CBr_2_ generated from CHBr_3_. New peaks appeared at binding energies of 71 and 182 eV in all of the samples. These were assigned to Br 3d and Br 3p signals, respectively [24–25]. Figure 2b shows the results obtained from SAMs after modification with :CCl_2_ generated from CHCl_3_. New signals at binding energies of 201 and 270 eV were detected. These were assigned to Cl 2p and Cl 2s [26]. Finally Figure 2c represents the surface after chemical modification with :CF_2_ generated from TMSCF_3_. A new signal at binding energy of 688.7 eV was detected and assigned to F 1s, consistent with a CF_2_ group present on the surface [27].

**Scheme 1:**
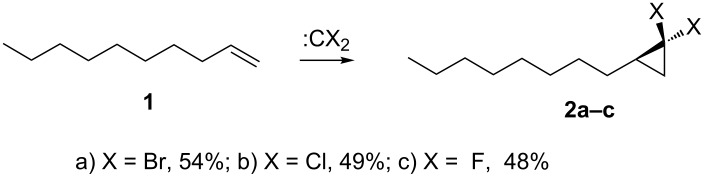
Model reactions of dec-1-ene (**1**) with dihalocarbenes in the liquid phase. a) and b) NaOH, BTEAC, CHX_3_, DCM, 8 h, 25 °C; c) TMSCF_3_, THF, NaI, 3 h, 65 °C.

It was anticipated that *gem*-dibromo-, *gem*-dichloro- and *gem*-difluorocyclopropane-terminated SAMs will be formed, following the usual transformations of these carbenes with double bonds. To add further support to this expectation, model reactions were carried out under each of the reaction conditions with dec-1-ene (**1**, Scheme 1). All of the cyclopropane products **2a-c** were obtained cleanly and in moderate yields (see Supporting Information File 1). The results of the model reactions demonstrate that formation of the dihalocyclopropane rings is a relatively clean process for this long chain terminal vinyl substrate. The absence of any side products gives confidence that only dihalocyclopropanes will be formed in the surface reactions.

Turning to the C_11_-vinyl-terminated SAMs products. In each case the presence of *gem*-dihalocyclopropane groups on the surface is supported by the ratios of the C 1s signals to the Br 3d, Cl 2p or F 1s signal, respectively. The theoretical and experimental ratios between the carbon and halogen XPS signals are summarised in Table 1. In all cases the ratios are consistent with a modification coverage of ~30%, with a slightly lower conversion rate in case of F, which might be due to its higher electronegativity and an associated higher repulsion between the terminal groups after cyclopropanation. Conversion rates were determined by correcting the experimental C3/(C1 + C2) ratios from Table 1 with a factor of *d*/(λ(1 *−* exp(−*d*/λ)), where *d* is the film thickness (determined with ellipsometry) and λ the mean free path of the electrons. This accounts for the partial attenuation of the C3 XPS carbon signal. The water contact angles (CAs) of Br, Cl and F carbene treated surfaces were recorded and the CA values obtained of 80°, 85° and 104°, respectively, are in good agreement with the literature [28–30]. Notably the fluorinated SAM has the largest contact angle as expected, however, the increase and the final contact angle values are clearly lower than that for a pure CF_3_ terminated film (~118–120°) [31], but this is not surprising given that the halogen functional group density is lower.

**Table 1:** Assignment of the C 1s XPS signals after treatment of C_11_-vinyl SAMs with the respective dihalocarbene. Theoretical and experimental ratios of the Br 3d to C 1s, Cl 2p to C 1s and F 1s to C 1s XPS signals of modified C_11_-vinyl SAMs.

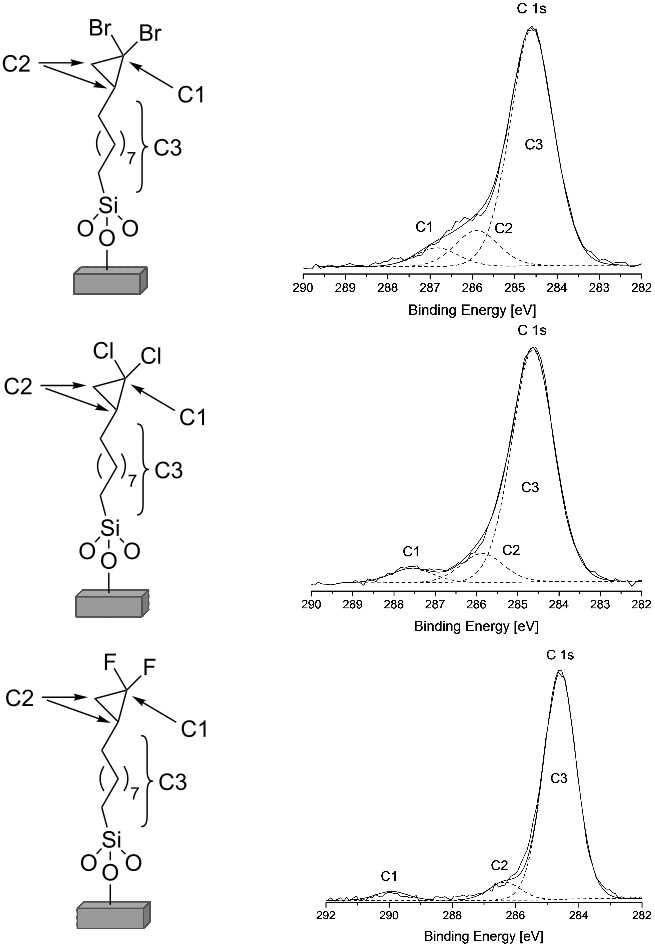					

		Ratios			
		X:C1	X:C2	C1:C2	C3:(C1 + C2)

Theor.	:CX_2_	2:1	1:1	1:2	3:1
Exp.	:CBr_2_	1.9:1	0.9:1	1:2.1	7.6:1
Exp.	:CCl_2_	2.2:1	1.1:1	1:2	7.4:1
Exp.	:CF_2_	2.1:1	1:1	1:2	8.5:1

**Figure 3:**
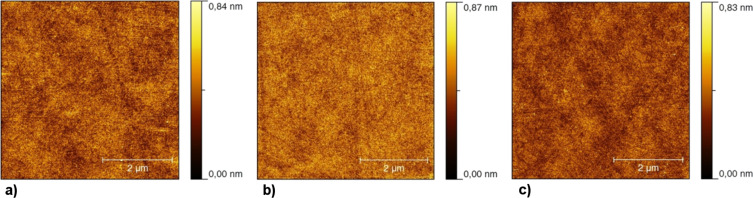
AFM images of 5 μm × 5 μm area of C_11_-vinyl SAMs modified with a) :CBr_2_ carbene, RMS 93 pm; b) :CCl_2_ carbene, RMS 101 pm; c) TMSCF_3_, RMS 79 pm.

Finally AFM images were recorded for the three dihalocyclopropane modified surfaces and they are shown in Figure 3a–c. In all cases the images are smooth and defect free. There was no excess of material observed from reagents after washing, and in each case the RMS surface roughness values did not exceeded 150 pm. It is clear that there is no detectable change observed in the film after each modification, and that the films are of good integrity.

An alternative approach, also illustrated in Figure 1 is to prepare SAMs using pure samples of pre-halogenated cyclopropyl chains, with deposition directly onto the surface. This presents the obvious challenge of obtaining highly ordered films after direct deposition. The current approach establishes films of good integrity, which then become chemically modified. There is good evidence that this is less straightforward with functionalised surfactants [32].

## Conclusion

In summary we have been able to demonstrate that vinyl-terminated SAMs can be chemically modified by a range of dihalocarbenes to generate surfaces carrying the corresponding dihalocyclopropane motifs. The reactions demonstrate that these organic chemical transformations, which have been relatively widely used in solution reactions of olefins, can be extended to surface reactions of SAMs. This opens up prospects too of modifying surfaces in this manner with carbenes carrying more elaborate functional groups, and thus a more dramatic change to the surface properties.

## Experimental

Bromoform (CHBr_3_), chloroform (CHCl_3_) and the Ruppert–Prakash reagent (CF_3_Si(CH_3_)_2_) [33–34] were used as the carbene precursors for surface modification, with the resultant carbenes generated in solution. For dibromo- and dichlorocarbene generation a solution of NaOH with CHBr_3_ or CHCl_3_ was stirred with a solution of benzyltriethylammonium chloride (BTEAC, 0.1 mmol) in dichloromethane for 10 min at 0 °C. Pre-coated silicon wafers (1 cm × 1.5 cm) with C_11_-vinyl-terminated SAMs, were immersed in the reaction mixture and the liquids were stirred at room temperature for fixed periods of time (see Supporting Information File 1). SAMs on silicon substrates form stable films [6–7], however, they can be vulnerable to chemical degradation particularly in aqueous base [28,35–36]. For this reason the NaOH concentration and reaction time required to be optimised. The reaction temperature was kept at 25 °C and the phase-transfer catalyst, benzyltriethylammonium chloride (BTEAC) was chosen to generate the :CX_2_ carbenes, and minimise exposure of the wafers to the base.

**Figure 4:**
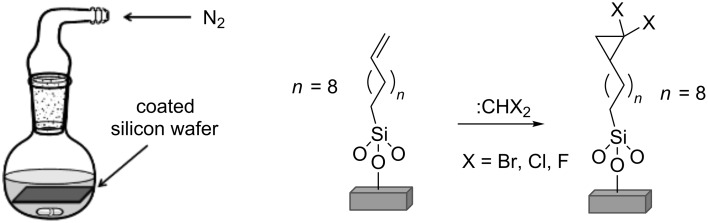
The experimental set-up for the dibromo-, dichloro- and difluorocarbene reactions performed on C_11_-vinyl SAMs.

The Ruppert–Prakash reagent (TMSCF_3_) was used to prepare the *gem*-difluorocyclopropane-terminated SAMs by generating difluorocarbene, following the procedure of Wang et al. [22] for small molecule transformations. This involved stirring a solution of NaI (0.2 equiv) and TMSCF_3_ in THF (2 mL), and then immersing the silicon wafers (1 cm × 1.5 cm) into the reaction mixture at 65 °C for a fixed period of time (see Supporting Information File 1). The experimental set-up for the surface modification with the three different carbenes is shown in Figure 4. Details of the surface analytical techniques used are given in the Supporting Information File 1.

## Supporting Information

File 1 Synthesis protocols and additional surface analysis data.
