# Supplementary material for: Organic chemistry on surfaces: Direct cyclopropanation by dihalocarbene addition to vinyl terminated self-assembled monolayers (SAMs)
Source: Beilstein J Org Chem. 2014 Dec 5;10:2897–902. doi: 10.3762/bjoc.10.307 (PMC4273226; doi:10.3762/bjoc.10.307)
Supplement: File 1 — Synthesis protocols and additional surface analysis data. [file Beilstein_J_Org_Chem-10-2897-s001.pdf]

Supporting Information

for

Organic chemistry on surfaces: Direct cyclopropanation by dihalocarbene addition to vinyl terminated self-assembled monolayers (SAMs)

Malgorzata Adamkiewicz, David O'Hagan* and Georg Hähner*

Address: EaStCHEM School of Chemistry, University of St. Andrews, St. Andrews, Fife KY16 9ST, UK

*Corresponding author

Email: David O'Hagan - do1@st-andrews.ac.uk, Georg Hähner - gh23@st-andrews.ac.uk

Synthesis protocols and additional surface analysis data

SYNTHESIS

Synthesis of 1,1-dichloro-2-octylcyclopropane (2b)

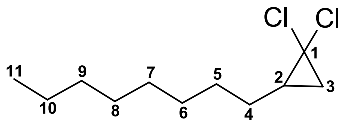

Sodium hydroxide (3.0 g, 75 mmol, 12 equiv) and BTEAC (0.02 g, 0.1 mmol) were added to dichloromethane (6 mL) at room temperature. The mixture was cooled to 0 °C and then chloroform (6 mL) was added in a single portion. The reaction mixture was stirred for 10 min, then dec-1-ene (**1**, 0.88 g, 6.3 mmol, 1 equiv) was added dropwise over a period of 30

min. The mixture was stirred for 8 h at rt and then quenched by the addition of water (20 mL). The aqueous layer was extracted into dichloromethane (3 × 10 mL), and the combined organic extracts were dried over $MgSO_4$. The product was purified by silica gel chromatography (hexane), affording 1,1-dichloro-2-octylcyclopropane (**2b**, 0.69 g, 49%) as a colourless oil. ^{1}H NMR (400 MHz, $CDCl_3$) δ_H 1.63-1.40 (6H, m), 1.37-1.21 (10H, m), 1.06-1.01 (1H, m), 0.88 (3H, t, *J* 6.6 Hz, C$\underline{H}_3$) ^{13}C NMR (100 MHz, $CDCl_3$) δ_C 32.0, 31.0, 30.5, 29.6, 29.4, 29.3, 28.7, 26.9, 22.8, 14.3; *HRMS m/z* (CI): calculated for $C_{11}H_{20}{}^{35}Cl_2$ [M^+] 222.0937, found 222.0938.

Synthesis of 1,1-dibromo-2-octylcyclopropane (2a)

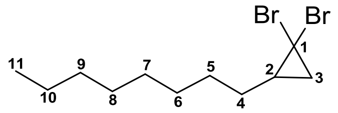

Sodium hydroxide (3.0 g, 75 mmol, 12 equiv) and BTEAC (0.02 g, 0.1 mmol) were added to dichloromethane (6 mL) at room temperature. The mixture was cooled to 0 °C and then bromoform (6 mL) was added in a single portion. The reaction mixture was stirred for 10 min, then dec-1-ene (**1**, 0.88 g, 6.3 mmol, 1 equiv) was added dropwise over a period of 30 min. The mixture was stirred for 8 h at rt and then quenched by the addition of water (20 mL). The aqueous layer was extracted into dichloromethane (3 × 10 mL), and the combined organic extracts were dried over $MgSO_4$. The product was purified by silica gel chromatography (hexane), affording 1,1-dibromo-2-octylcyclopropane (**2a**, 1.05 g, 54%) as a colourless oil. ^{1}H NMR (400 MHz, $CDCl_3$) δ_H 1.78-1.68 (1H, m), 1.66-1.41 (6H, m), 1.39-1.23 (10H, m), 0.88 (3H, t, *J* 6.5 Hz, C$\underline{H}_3$) ^{13}C NMR (100 MHz, $CDCl_3$) δ_C 32.6, 31.9, 31.5, 29.8, 29.5, 29.3, 29.2, 28.5, 28.3, 22.7, 14.1; *HRMS m/z* (CI): calculated for $C_{11}H_{19}{}^{79}Br_2$ $[M-H]^+$ 308.9848, found 308.9845.

Synthesis of 1,1-difluoro-2-octylcyclopropane (2c)

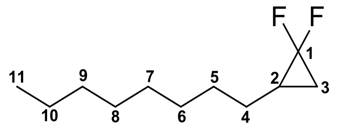

Sodium iodide (0.64 g, 4.3 mmol, 0.2 equiv), dec-1-ene (**1**, 3.0 g, 21.4 mmol, 1 equiv) and THF (40 mL) were placed in a round-bottom flask. $TMSCF_3$ (7.6 mL, 53.5 mmol, 2.5 equiv) was then added. The reaction mixture was heated at 65 °C for 2 h and then concentrated in vacuo. The crude was extracted into diethyl ether (3 × 25 mL), and the combined organic layers were washed sequentially with water (25 mL), sodium sulphite solution (25 mL), saturated sodium bicarbonate solution (25 mL), dried over $MgSO_4$ and then the solvent was removed in vacuo. The product was purified over silica gel (hexane), to afford 1,1-difluoro-2-octylcyclopropane (**2c**, 1.9 g, 48%) as a colourless oil; ^{1}H NMR (400 MHz, $CDCl_3$) δ_H 1.52-1.18 (17H, m), 0.88

(3H, t, *J* 6.6 Hz, C*H*3); ^{13}C NMR (100 MHz, $CDCl_3$) δ_C 114.7 (1C, t, *J* 283.2, Hz $\underline{C}F_2$), 31.9, 29.4, 29.2, 29.1, 28.8, 26.8, 26.7, 22.7-22.4 (1C, m), 15.9 (1C, t, *J* 11.2 Hz), 14.0; ^{19}F NMR (282 MHz, $CDCl_3$) δ_F -145.1 (1F, ddd, *J* 154.2, 12.8, 4.6 Hz), -128.1- (-128.7) (1F, m); *HRMS m/z* (CI): calculated for $C_{11}H_{19}F_2$ $[M-H]^+$ 189.1449, found 189.1444.

General experimental procedure for the reaction of $CHCl_3$ and $CHBr_3$ with SAMs

To a round-bottom flask (10 mL) equipped with a small stirring bar and cooled to 0 °C, NaOH (100 mg, 200 mg, 300 mg, 400 mg, 500 mg) and $CHCl_3$ or $CHBr_3$ (1 mL) were added. Next, a solution of benzyltriethylammonium chloride (BTEAC, 0.1 mmol) in dichloromethane (1 mL) was added and the reaction mixture was stirred for 10 min at 0 °C. Next, the silicon wafers (1 cm × 1.5 cm), pre-coated with vinyl-terminated SAMs, were immersed in the reaction mixture and the liquids were stirred at room temperature for a fixed period of time (30 min, 1 h, 2 h, 3 h, 4 h, 5 h). After the reaction, the wafers were washed several times with distilled water and then sonicated sequentially in dichloromethane, toluene and DI water for a minimum of 15 min in each solvent. Then the wafers were dried under a nitrogen atmosphere and stored in a desiccator until they have been characterised.

General experimental procedure for the reaction of $TMSCF_3$ with SAMs

To a round bottom flask (10 mL) equipped with a stirring bar, NaI (0.2 equiv) and $TMSCF_3$ (0.6 mM, 1.0 mM, 1.4 mM, 1.7 mM, 2.7 mM) in THF (2 mL) were added. Next, the silicon

wafers (1 cm × 1.5 cm), pre-coated with vinyl-terminated SAMs, were immersed in the reaction mixture and the solution was stirred at 65 °C for fixed periods of time (2 h, 3 h, 4 h, 5 h). After the reaction, the wafers were washed several times with distilled water and then sonicated sequentially in dichloromethane, toluene and DI water for a minimum of 15 min in each solvent. Then the wafers were dried under a nitrogen atmosphere and stored in a desiccator until characterised.

SURFACE ANALYSIS

XPS

X-ray photoelectron spectroscopy analysis was performed at the University of Newcastle (NEXUS at nanoLAB) and at the University of Edinburgh.
Newcastle X-ray photoelectron spectroscopy spectra were obtained using a K-Alpha instrument (Thermo Scientific) and Al Kα radiation (1486.6 eV). During the analysis, the pressure in the instrument chamber was kept around 3×10^{-8} mbar. The detector had a take-off angle of 90° relative to the surface. Survey spectra were recorded with the analyser pass energy set to 200 eV. Single region scans were recorded with the pass energy of the analyser set to 20 eV.
Edinburgh X-ray photoelectron spectroscopy spectra were obtained using a VG Sigma Probe (VG Scientific Ltd., UK) and Al Kα radiation (1486.6 eV). During the analysis, the pressure in the instrument chamber was kept around 1.33×10^{-8} mbar. The detector had a take-off angle of 37° relative to the surface. Survey spectra were recorded with the analyser pass energy set to 80 eV. The number of single region scans recorded for each element was typically 20, with the pass energy of the analyser set to 10 eV.

The XPS spectra were corrected for charging by referencing the aliphatic C 1s peak of hydrocarbons to 284.6 eV. Elemental compositions of the various surfaces were determined from the area under individual elemental peaks using sensitivity factors provided with the software as well as taking the transmission function of the analyser into account. CasaXPS (Casa Software Ltd., UK) was used for the analysis. The spectra were fitted using Gaussian/Lorentzian peak shapes with a ratio of 70%/30%. A Shirley background was subtracted for the quantitative analysis.

Contact angle analysis

Water contact angles (DI water) were measured on a G10 goniometer microscope (KRÜSS GmbH, Hamburg, Germany) under ambient conditions at room temperature. Droplets of ~3 μL were dispensed from a microburette. Reported values are the average of three measurements taken from different places of the surface. The error based on the variation of the contact angle of the organic films prepared under identical conditions was ± 1°.

AFM

The atomic force microscope used is a PicoSPM II (Molecular Imaging, AZ, USA) instrument with an interchangeable nose scanner. The nominal spring constant of the cantilevers used was 0.06–0.12 N/m (Veeco, CA, USA). Images were recorded in ambient in contact mode at a scanning speed of ~0.8 lines/s and with a nominal constant force on the order of 10 nN.

Elipsometry

The thickness of the SAMs was measured with an M-2000DI™ spectroscopic ellipsometer (J. A. Woollam Co., Inc., USA). Thickness values were extracted from fits to the data taken from 45 to 70° in steps of 5° over wavelengths from 200 to 1000 nm. The sample surface was modelled as a Si substrate with an oxide layer and a Cauchy layer. The thickness of the silicon oxide after the oxidative cleaning treatment was 16 ± 1 Å (average of three samples). The thickness of the monolayer films was calculated with a refractive index of 1.45. The error based on the observed variation of the thickness of the organic films prepared under identical conditions was ~2 Å.

SAM modification with $CHCl_3$ and $CHBr_3$

The results obtained on film thickness and contact angles before and after the reaction of vinyl- and methyl-terminated SAMs with $CHCl_3$ and $CHBr_3$. Each value is an average of measurements taken from two samples. The errors are based on standard deviation.

Precursor SAM	Carbene Precursor	NaOH [mg]	Time [h or min]	Film Thickness [Å]		Film Contact Angle [°]	
				Before	After	Before	After
C_{11}-vinyl	$CHCl_3$	100	30 min	~15	14.9±0.1	101±1	100±1
C_{11}-vinyl	$CHCl_3$	200	30 min	~15	15.1±0.1	101±1	98±1
C_{11}-vinyl	$CHCl_3$	300	30 min	~15	15.4±0.2	101±1	90±1
C_{11}-vinyl	$CHCl_3$	400	30 min	~15	12.9±0.1	101±1	87±1
C_{11}-vinyl	$CHCl_3$	500	30 min	~15	9.7±0.2	101±1	79±1
C_{11}-vinyl	$CHBr_3$	100	30 min	~15	15.6±0.1	101±1	101±1
C_{11}-vinyl	$CHBr_3$	200	30 min	~15	15.4±0.2	101±1	93±1
C_{11}-vinyl	$CHBr_3$	300	30 min	~15	14.9±0.3	101±1	92±1
C_{11}-vinyl	$CHBr_3$	400	30 min	~15	10.2±0.4	101±1	84±1
C_{11}-vinyl	$CHBr_3$	500	30 min	~15	9.2±0.1	101±1	80±1
C_{11}-vinyl	$CHCl_3$	300	1 h	~15	15.0±0.2	101±1	93±1
C_{11}-vinyl	$CHCl_3$	300	2 h	~15	14.8±0.3	101±1	90±1
C_{11}-vinyl	$CHCl_3$	300	3 h	~15	16.4±0.1	101±1	85±1
C_{11}-vinyl	$CHCl_3$	300	4 h	~15	13.7±0.1	101±1	82±1
C_{11}-vinyl	$CHCl_3$	300	5 h	~15	9.5±0.2	101±1	75±1
C_{11}-vinyl	$CHBr_3$	300	1 h	~15	15.2±0.1	101±1	91±1
C_{11}-vinyl	$CHBr_3$	300	2 h	~15	16.1±0.2	101±1	82±1
C_{11}-vinyl	$CHBr_3$	300	3 h	~15	16.6±0.3	101±1	80±1
C_{11}-vinyl	$CHBr_3$	300	4 h	~15	11.6±0.4	101±1	72±1
C_{11}-vinyl	$CHBr_3$	300	5 h	~15	9.9±0.1	101±1	70±1
C_{15}-vinyl	$CHCl_3$	300	3 h	~19	19.6±0.2	105±1	85±1
C_{15}-vinyl	$CHBr_3$	300	3 h	~19	19.1±0.3	105±1	80±1
C_{18}-methyl	$CHCl_3$	300	3 h	~26	26.2±0.1	109±1	109±1
C_{18}-methyl	$CHBr_3$	300	3 h	~26	25.6±0.3	109±1	109±1

SAM modification with $TMSCF_3$

The results obtained on film thickness and contact angles before and after the reaction of vinyl- and methyl-terminated SAMs with $TMSCF_3$. Each value is an average of measurements taken from two samples. The errors are based on standard deviation.

Precursor SAM	$TMSCF_3$ [mM]	Time [h]	Film Thickness [Å]		Film Contact Angle [°]	
			Before	After	Before	After
C_{11}-vinyl	0.6	3 h	~15	15.6±0.2	101±1	101±1
C_{11}-vinyl	1.0	3 h	~15	15.7±0.3	101±1	101±1
C_{11}-vinyl	1.4	3 h	~15	16.1±0.1	101±1	103±1
C_{11}-vinyl	1.7	3 h	~15	17.1±0.1	101±1	104±1
C_{11}-vinyl	2.7	3 h	~15	14.0±0.2	101±1	100±1
C_{11}-vinyl	1.7	2 h	~15	16.0±0.3	101±1	103±1
C_{11}-vinyl	1.7	3 h	~15	17.5±0.1	101±1	104±1
C_{11}-vinyl	1.7	4 h	~15	13.9±0.2	101±1	103±1
C_{11}-vinyl	1.7	5 h	~15	13.4±0.3	101±1	100±1
C_{11}-vinyl	1.7	48 h	-	-	101±1	83±1
C_{15}-vinyl	1.7	3 h	~19	19.2±0.1	103±1	104±1
C_{18}-methyl	1.7	3 h	~26	26.0±0.2	109±1	109±1
